# Multiple Myeloma Macrophages: Pivotal Players in the Tumor Microenvironment

**DOI:** 10.1155/2013/183602

**Published:** 2013-01-30

**Authors:** Simona Berardi, Roberto Ria, Antonia Reale, Annunziata De Luisi, Ivana Catacchio, Michele Moschetta, Angelo Vacca

**Affiliations:** Department of Biomedical Sciences and Human Oncology, Clinica Medica “G. Baccelli,” University of Bari Aldo Moro Medical School, Piazza Giulio Cesare 11, 1-70124 Bari, Italy

## Abstract

Tumor microenvironment is essential for multiple myeloma (MM) growth, progression, and drug resistance through provision of survival signals and secretion of growth and proangiogenic factors. This paper examines the importance of macrophages within MM bone marrow (BM) microenvironment, referred to as MM-associated macrophages, as a potential niche component that supports tumor plasma cells. These macrophages are derived from peripheral blood monocytes recruited into the tumor. Upon activation by MM plasma cells and mesenchymal stromal cells, macrophages can release growth factors, proteolytic enzymes, cytokines, and inflammatory mediators that promote plasma cell growth and survival. Macrophages promote tumor progression through several mechanisms including angiogenesis, growth, and drug resistance. Indeed, these macrophages are essential for the induction of an angiogenic response through vasculogenic mimicry, and this ability proceeds in step with progression of the plasma cell tumors. Data suggest that macrophages play an important role in the biology and survival of patients with MM, and they may be a target for the MM antivascular management.

## 1. Tumor-Associated Macrophages

In the past decades, the major focus of cancer research has been the malignant cell itself. In haematological malignancies, including multiple myeloma (MM), this has led to the identification of molecular alterations affecting growth control and apoptotic pathways [1]. Recent studies add yet another facet to the complex multistep model of tumorigenesis by demonstrating that tumor cells carrying genomic and epigenomic abnormalities also trigger changes in their microenvironment [2]. Indeed, accumulating evidence supports the hypothesis that the tumor microenvironment or “niche” ultimately determines the clinical behavior of the disease and has direct impact on overall prognosis [3]. 

MM is characterized by the accumulation of monoclonal plasma cells in the bone marrow (BM) where they grow and expand. This suggests the importance of the BM microenvironment in supporting MM cell growth and survival [4]. The roles of BM stromal cells in supporting MM plasma cells have been extensively studied. The interaction between plasma cells and stromal cells confers plasma cell homing, growth, survival, and resistance to chemotherapy [5]. Among stromal cells, the inflammatory cells play an indispensable role in disease progression [6]. Within the tumor stroma, the macrophage is the pivotal member of inflammatory cells. Tumor-associated macrophages (TAMs), which constitute a significant part of the tumor infiltrating immune cells, have been linked to the growth, angiogenesis, and metastasis of a variety of cancers [7]. In MM, macrophages are an abundant and important component of the stromal cells, contributing to tumor angiogenesis [8] in line with several reports describing an association between macrophage infiltration, vascularity, and prognosis [9]. 

## 2. Macrophage Activation and Polarization

Macrophages constitute an extremely heterogeneous population originating from blood monocytes, that are capable of displaying different functional activities, some of which are antagonistic; for instance, they can be immunostimulatory or immunosuppressive and either promote or restrain inflammation [10]. This functional plasticity is regulated by local cues to which the macrophages respond. Macrophage heterogeneity has been simplified in cell polarization concept that discriminates macrophages into distinct types, schematically identified as M1 (or “classically activated”) and M2 (or “alternatively activated”). In general, M1 macrophages are stimulated by bacterial products and cytokines secreted by Th1 cells; they act as soldiers defending the host from viral and microbial infections, fighting against tumors, producing high amounts of inflammatory cytokines and activating immune response [11]. On the other hand, distinct types of M2 cells differentiate when monocytes are stimulated with interleukin-4 (IL-4) and IL-13 or with IL-10 and glucocorticoids [12]. M2 macrophages are characterized by poor antigen-presenting capability and wound-healing promotion [13]. Further, these macrophages express specific change in some metabolic pathways; arginine metabolism is oriented toward the production of ornithine and polyamine instead of citrulline and nitric oxide. M2 cells are workers of the host; they promote scavenging of debris, angiogenesis, remodeling, and repair of wounded/damaged tissues. Of note, M2 cells control the inflammatory response by downregulating M1 cell-mediated functions [14]. TAMs (including MM-associated macrophages) resemble M2-like macrophage population with little cytotoxicity for tumor cells because of their limited production of nitric oxide and proinflammatory cytokines [15]. TAMs also possess poor antigen-presenting capability and effectively suppress T cell activation. In the majority of cancers, TAMs show mostly protumoral functions, promoting tumor cell survival, proliferation, and dissemination by secreting a wide range of growth and proangiogenic factors as well as metalloproteinases, and by their involvement in signalling circuits that regulate the function of fibroblasts in the tumor stroma [7]. 

## 3. Current Concepts of MM-Associated Macrophages

In patients with active (symptomatic) MM, fluorescence-activated cell sorting (FACS) analysis on freshly isolated BM mononuclear cells revealed higher percentages of CD68^+^ macrophages (a glycoprotein expressed only by human macrophages) than in patients with nonactive disease (i.e., in partial/complete remission, or in plateau phase) or those with monoclonal gammopathies of undetermined significance (MGUS). MGUS is a premalignant, asymptomatic disorder characterized by monoclonal plasma cell proliferation in BM with absence of end-organ damage that represents a benign plasma cell disorder. Histologically, in patients with active MM, CD68^+^ macrophages were heavily infiltrated in the BM. Indeed, in these patients, macrophages are recruited from the BM pool and/or the circulation into the vascular endothelial growth factor (VEGF) plus fibroblast growth factor-2- (FGF-2-) rich microenvironment [16], both factors being chemotactic for macrophages. Scavelli et al. demonstrated that BM macrophages in patients with active MM are functionally, phenotypically, and morphologically different from those of patients with nonactive disease and MGUS [8]. Indeed, macrophages of these patients are similar to paired endothelial cells (MMECs) and contribute to angiogenesis through vasculogenic mimicry, in parallel to progression of plasma cell tumours [17]. It may well be that in active MM, plasma cells secrete VEGF and FGF-2 that induce inflammatory cells to secrete their own VEGF, FGF-2, and hepatocyte growth factor (HGF); all these cytokines continuously recruit and activate MM-associated macrophages to adapt functionally, phenotypically, and morphologically to become vicarious MMECs, mimicking these cells, and collaborating with them in vessel formation [18]. This is likely minimal in nonactive MM or cannot take place in MGUS or benign anemia patients, due to the absence or small number of plasma cells, hence, very low levels of secreted VEGF and FGF-2, as previously demonstrated [19].

Moreover, BM macrophages protect MM cells from spontaneous and melphalan-induced apoptosis [20]. However, the exposure of macrophages in MM during the treatment with zoledronic acid and bortezomib, alone and/or in combination, impacts their angiogenic and vasculogenic properties, suggesting that these cells may be considered as a target of both drugs in MM patients. These findings indicate that macrophages (as TAMs) may be an abundant and important component of the BM stromal cells and play a critical role in MM tumor progression.

## 4. The Role of MM-Associated Macrophages in Tumor Progression (Figure 1)

### 4.1. Growth Promoting Properties of MM-Associated Macrophages

Macrophage infiltration positively correlates with MM cell survival and proliferation. Indeed, MM macrophages are characterized by higher expression of factors that stimulate plasma cell proliferation and survival, including IL-6 and IL-10, and lower expression of IL-12 and tumor necrosis factor-alpha (TNF-*α*) [21]. It has been shown that IL-10 stimulates the proliferation of MM cells freshly isolated from patients in IL-6 deprived cultures [22]. Additionally, both IL-12 and TNF-*α* are considered to retain antitumor effects [23]; hence, a lower expression of these cytokines by macrophages could provide a favourable milieu for the growth of malignant cells. Interestingly, MM macrophages have increased levels of VEGFA and VEGFC mRNA expression [21]. It is well known that VEGFs play a critical role in MM pathology by their effect on vascular endothelial cells, one of the well-known components of the MM plasma cell niche [24]. Traditionally, it has been assumed that mesenchymal stromal cells (MSCs) are the major source of VEGFs [25], but current results suggest the interesting finding that macrophages might be another major contributor of VEGFs, especially when they have been educated by MSCs. Based on an *in vivo* model of MSCs transplantation into rat hind limb ischemia model, the source of increased VEGF in the tissues was found to be not transplanted (human) MSCs but recipient (rat) macrophages [26]. 

### 4.2. Angiogenesis Promoting Properties of MM-Associated Macrophages

BM neovascularization is a constant hallmark of MM, but not of MGUS. This phenomenon forms partly through angiogenesis [18] and is endowed with the overangiogenic phenotype of MMECs [27]. Mature macrophages have been found to form capillary-like lumina and branching patterns *in vitro*, participating to *de novo* formation of microvessels [28]. Scavelli et al. demonstrated that BM macrophages in patients with active MM contribute to build neovessels through vasculogenic mimicry, in parallel to progression of plasma cell tumors [8]. Macrophages from MM patients exposed to VEGF and FGF-2, which are major angiogenic cytokines secreted by plasma cells and present in the BM microenvironment, transformed into cells functionally and phenotypically similar to paired MMECs, generating capillary-like networks mimicking those of MMECs. Macrophages from nonactive MM, MGUS, and benign anemia patients displayed similar, albeit weaker, features [8]. EC-like macrophages and apparently typical macrophages contributed sizably to form the neovessel wall in patients with active MM, whereas their vascular supply was minimal in nonactive MM and absent in MGUS patients. These data suggest that in active MM, macrophages contribute to neovascularization through a vasculogenic pathway, and that in nonactive MM and MGUS, they are prone to behave accordingly, marching in step with progression, hence, with the vascular switch [29]. MM-associated macrophages present morphological differences from those from nonactive MM or MGUS and benign anemia patients; they displayed oblong and spindle shape with thin cytoplasmic extroversions, some of which were either arranged to form a microvessel-like lumen or anastomosed with each other and with those of nearby macrophages to form tube-like structures. In contrast, macrophages from the other patients' groups were rounded in shape and gave no extroversions or only rare, short ones. These differences could be due to higher levels of VEGF and FGF-2 in the BM milieu of active MM [16], hence, to an intense, continuous paracrine stimulation of cells, as occurs in paired MMECs [27]. Under VEGF plus FGF-2 stimulation, MM macrophages undergo a phenotypic and functional adaptation [30], starting to behave like MMECs, expressing typical markers of paired MMECs that are FVIII-RA, VEGFR-2, and VE-cadherin, and retaining their own CD14 and CD68 markers. Macrophages of nonactive MM, MGUS, and benign anemia patients exposed to VEGF plus FGF-2 underwent morphological, phenotypic, and functional changes indicative of vascular mimicry, becoming prone to form neovessels [8]. The vasculogenic switch by macrophages may be induced by the numerous VEGF and FGF-2 secreting plasma cells in the active MM and emerges with progression from MGUS to MM. VEGF and FGF-2 may act via their respective binding to VEGFR-1, the only VEGF receptor present on macrophages [31], and the FGF-2 receptors FGFR-1/-2/-3. VEGFR-1 mediates macrophage chemotaxis [31] and the organization of the embryo vasculature by vasculogenesis [32], but not the definitive vessel assembly, which is closely dependent on VEGFR-2, a specific EC differentiation marker [33].

Exposure of active MM macrophages to VEGF plus FGF-2 leads to an increase in the expression of Tie2/Tek and VEGFR-2, and a slight decrease in FGFR-2, all at levels overlapping those of paired MMECs. The intense expression of VEGFR-2 and Tie2/Tek, together with the decreased expression of VE-cadherin, a specific inter-EC adhesion molecule, is indicative of ongoing neovascularization [8]. In patients with active MM, FACS analysis on freshly isolated BM mononuclear cells revealed higher percentages of CD14/CD68 double-positive cells than in patients with nonactive disease and with MGUS.

Since BM macrophages from patients with active MM keep their CD14 and CD68 lineage markers, they can be regarded as cells that do not transdifferentiate into ECs, but adapt functionally, phenotypically, and morphologically to be like MMECs. The EC-like macrophages are morphologically and histochemically similar to sinusoid-lining cells of human lymphoid tissue, a special subset of macrophages that express FVIII-RA [34]. The behaviour of these macrophage types in active MM can thus be regarded as a “vasculogenic mimicry,” like that of melanoma and other tumor cells which form vascular channels to cater for their rapid proliferation and high need of vessels [35]. Moreover, MM macrophages synthesize and release inducible nitric oxide synthase, which increases blood flow and promotes angiogenesis [17]. 

### 4.3. Immunosuppressive Properties of MM-Associated Macrophages

TAMs promote tumor growth not only by supporting angiogenesis but also by inducing immunosuppression [36].

In MM, recent evidence attributes a major role in immunosuppression to myeloid-derived suppressor cells (MDSCs) [37]. 

MDSCs represent a heterogeneous population of immature myeloid cells that lack in the expression of cell surface markers specifically expressed by monocytes, macrophages, or dendritic cells and with a potent suppressive effect on T cells. MDSCs are phenotypically characterized by CD14− CD11b+ or CD33+, which is a common marker for myeloid cells, and lack in markers for mature myeloid and lymphoid cells such as HLA-DR [38]. 

MDSCs are significantly increased in patients with MM compared to patients with MGUS and healthy controls, as a consequence of factors associated with inflammation, such as increased secretion of VEGF, IL-1*β*, IL-6, and prostaglandin E2 [37]. 

MDSCs play their immunosuppressive activity through various mechanisms encompassing arginase, inducible nitric oxide synthase, and reactive oxygen species [38]. Arginase-1 and nitric oxide synthase-2, released by MDSCs, are key enzymes in L-arginine catabolism, which work synergistically in inhibiting T cell proliferation and MHC II expression and in promoting apoptosis. Moreover, arginase-1 activation mediates H_2_O_2_ production by MDSCs that inhibit the release of IFN-*γ*, essential for the stimulation of naïve T cell differentiation and, hence, for the promotion of immune evasion [38]. 

Serafini et al. demonstrated the ability to use clinically available phosphodiesterase-5 (PDE5) inhibitors to overcome the MDSC-mediated immunosuppressive pathway in MM. PDE5 blockade in MDSCs from MM patients downregulates IL-4R*α* expression which is correlated with L-arginine expression. These data suggest the use of PDE5 inhibitors as therapeutically effective drugs to overcome tumor-induced immunosuppression [39]. 

### 4.4. Role of MM-Associated Macrophages in Chemotherapy Resistance

Although chemotherapy is now the most effective treatment for MM, plasma cells often fail to respond to the drugs. Studies have shown that the response of MM plasma cells to cytotoxic chemotherapeutics can be attenuated by the presence of BM stromal cells [40]. Coculture of MM plasma cells with macrophages protected plasma cells from melphalan-induced apoptosis by inhibiting the activation and cleavage of caspase-3 and poly(ADP-ribose) polymerization (PARP) and maintaining the levels of Bcl-XL. These results suggest that macrophages protect MM cells from apoptosis via inhibiting Bcl-XL-dependent caspase activation [20]. 

### 4.5. Role of MM-Associated Macrophagesin as Therapeutic Target

Bortezomib (BZ) and zoledronic acid (ZOL) synergistically impact MM macrophage proliferation, adhesion, and migration, as well as VEGF, FGF-2, HGF, and PDGF secretion [21]. These drugs synergistically inhibit macrophage vasculogenesis on Matrigel and the expression of FVIII-RA, Tie2/Tek, and VEGFR-2/VE-cadherin, indicative of cell transdifferentiation into EC-like cells. Both drugs reduce phosphoactivation of VEGFR-2 and ERK1/2 and NF-KB activity. These data provide evidence that the exposure of BM macrophages during the treatment with BZ and ZOL impacts their angiogenic and vasculogenic properties, suggesting that these cells may be considered as a target of both drugs in MM patients.

## 5. Conclusions

The BM microenvironment plays a crucial role in the pathophysiology of MM. Substantial evidence suggests that MM-associated macrophages promote plasma cell growth and confer the ability to develop a vasculature which favours the disease progression. In summary, macrophages are key regulators of the angiogenic switch in MM, suggesting why the density of these cells is correlated with microvascular density and poor prognosis. Based on these findings, the development of antimacrophage therapeutics that target specific pathways associated with angiogenesis might contribute to the armamentarium of agents for treating MM or preventing the conversion of MGUS to active MM.

## Figures and Tables

**Figure 1:**
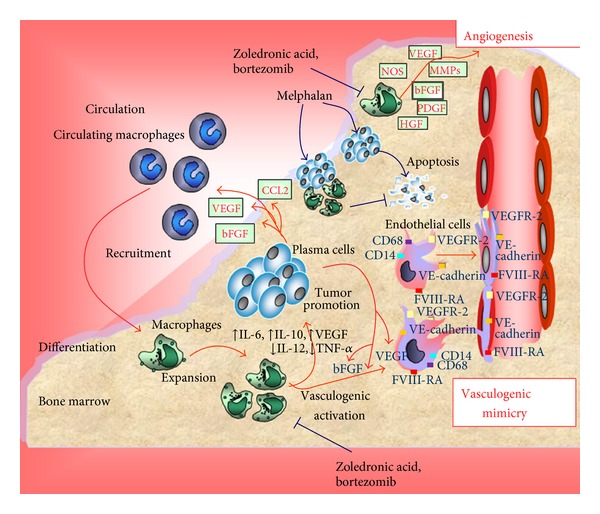
Role of MM-associated macrophages in BM microenvironment.

## References

[1] Kastrinakis NG, Gorgoulis VG, Foukas PG, Dimopoulos MA, Kittas C (2000). Molecular aspects of multiple myeloma. *Annals of Oncology*.

[2] Bissell MJ, Hines WC (2011). Why don’t we get more cancer? A proposed role of the microenvironment in restraining cancer progression. *Nature Medicine*.

[3] Dalton WS, Hazlehurst L, Shain K, Landowski T, Alsina M (2004). Targeting the bone marrow microenvironment in hematologic malignancies. *Seminars in Hematology*.

[4] Kyle RA, Rajkumar SV (2004). Multiple myeloma. *The New England Journal of Medicine*.

[5] Dalton WS (2003). The tumor microenvironment: focus on myeloma. *Cancer Treatment Reviews*.

[6] Coussens LM, Werb Z (2002). Inflammation and cancer. *Nature*.

[7] Pollard JW (2004). Tumour-educated macrophages promote tumour progression and metastasis. *Nature Reviews Cancer*.

[8] Scavelli C, Nico B, Cirulli T (2008). Vasculogenic mimicry by bone marrow macrophages in patients with multiple myeloma. *Oncogene*.

[9] Gendron RL, Tsai FY, Paradis H, Arceci RJ (1996). Induction of embryonic vasculogenesis by bFGF and LIF in vitro and in vivo. *Developmental Biology*.

[10] Martinez FO, Helming L, Gordon S (2009). Alternative activation of macrophages: an immunologic functional perspective. *Annual Review of Immunology*.

[11] Mantovani A, Sica A, Locati M (2007). New vistas on macrophage differentiation and activation. *European Journal of Immunology*.

[12] Mantovani A, Sica A, Sozzani S, Allavena P, Vecchi A, Locati M (2004). The chemokine system in diverse forms of macrophage activation and polarization. *Trends in Immunology*.

[13] Condeelis J, Pollard JW (2006). Macrophages: obligate partners for tumor cell migration, invasion, and metastasis. *Cell*.

[14] Mantovani A, Allavena P, Sica A (2004). Tumour-associated macrophages as a prototypic type II polarised phagocyte population: role in tumour progression. *European Journal of Cancer*.

[15] Mills CD, Kincaid K, Alt JM, Heilman MJ, Hill AM (2000). M-1/M-2 macrophages and the Th1/Th2 paradigm. *Journal of Immunology*.

[16] Ribatti D, Nico B, Vacca A (2006). Importance of the bone marrow microenvironment in inducing the angiogenic response in multiple myeloma. *Oncogene*.

[17] Jenkins DC, Charles IG, Thomsen LL (1995). Roles of nitric oxide in tumor growth. *Proceedings of the National Academy of Sciences of the United States of America*.

[18] Vacca A, Ribatti D, Presta M (1999). Bone marrow neovascularization, plasma cell angiogenic potential, and matrix metalloproteinase-2 secretion parallel progression of human multiple myeloma. *Blood*.

[19] Di Raimondo F, Azzaro MP, Palumbo GA (2000). Angiogenic factors in multiple myeloma: higher levels in bone marrow than in peripheral blood. *Haematologica*.

[20] Zheng Y, Cai Z, Wang S (2009). Macrophages are an abundant component of myeloma microenvironment and protect myeloma cells from chemotherapy drug-induced apoptosis. *Blood*.

[21] Kim J, Denu RA, Dollar BA (2012). Macrophages and mesenchymal stromal cells support survival and proliferation of multiple myeloma cells. *British Journal of Haematology*.

[22] Gu ZJ, Costes V, Lu ZY (1996). Interleukin-10 is a growth factor for human myeloma cells by induction of an oncostatin M autocrine loop. *Blood*.

[23] Li S, Zhang X, Xia X (2002). Regression of tumor growth and induction of long-term antitumor memory by interleukin 12 electro-gene therapy. *Journal of the National Cancer Institute*.

[24] Roccaro AM, Hideshima T, Raje N (2006). Bortezomib mediates antiangiogenesis in multiple myeloma via direct and indirect effects on endothelial cells. *Cancer Research*.

[25] Mayer H, Bertram H, Lindenmaier W, Korff T, Weber H, Weich H (2005). Vascular endothelial growth factor (VEGF-A) expression in human mesenchymal stem cells: autocrine and paracrine role on osteoblastic and endothelial differentiation. *Journal of Cellular Biochemistry*.

[26] Laurila JP, Laatikainen L, Castellone MD (2009). Human embryonic stem cell-derived mesenchymal stromal cell transplantation in a rat hind limb injury model. *Cytotherapy*.

[27] Vacca A, Ria R, Semeraro F (2003). Endothelial cells in the bone marrow of patients with multiple myeloma. *Blood*.

[28] Anghelina M, Moldovan L, Zabuawala T, Ostrowski MC, Moldovan NI (2006). A subpopulation of peritoneal macrophages form capillary-like lumens and branching patterns in vitro. *Journal of Cellular and Molecular Medicine*.

[29] Kumar S, Witzig TE, Timm M (2004). Bone marrow angiogenic ability and expression of angiogenic cytokines in myeloma: evidence favoring loss of marrow angiogenesis inhibitory activity with disease progression. *Blood*.

[30] Moldovan NI (2005). Functional adaptation: the key to plasticity of cardiovascular “stem” cells?. *Stem Cells and Development*.

[31] Barleon B, Sozzani S, Zhou D, Weich HA, Mantovani A, Marmé D (1996). Migration of human monocytes in response to vascular endothelial growth factor (VEGF) is mediated via the VEGF receptor flt-1. *Blood*.

[32] Fong GH, Rossant J, Gertsenstein M, Breitman ML (1995). Role of the Flt-1 receptor tyrosine kinase in regulating the assembly of vascular endothelium. *Nature*.

[33] Shalaby F, Ho J, Stanford WL (1997). A requirement for Flk1 in primitive and definitive hematopoiesis and vasculogenesis. *Cell*.

[34] Baroni CD, Vitolo D, Remotti D (1987). Immunohistochemical heterogeneity of macrophage subpopulations in human lymphoid tissues. *Histopathology*.

[35] Maniotis AJ, Folberg R, Hess A (1999). Vascular channel formation by human melanoma cells in vivo and in vitro: vasculogenic mimicry. *American Journal of Pathology*.

[36] Ben-Baruch A (2006). Inflammation-associated immune suppression in cancer: the roles played by cytokines, chemokines and additional mediators. *Seminars in Cancer Biology*.

[37] Brimnes MK, Vangsted AJ, Knudsen LM (2010). Increased level of both CD4+FOXP3+ Regulatory t Cells and CD14+HLA-DR^−^/low myeloid-derived suppressor cells and decreased level of dendritic cells in patients with multiple myeloma. *Scandinavian Journal of Immunology*.

[38] Gabrilovich DI, Nagaraj S (2009). Myeloid-derived suppressor cells as regulators of the immune system. *Nature Reviews Immunology*.

[39] Serafini P, Meckel K, Kelso M (2006). Phosphodiesterase-5 inhibition augments endogenous antitumor immunity by reducing myeloid-derived suppressor cell function. *Journal of Experimental Medicine*.

[40] Epstein J, Yaccoby S (2003). Consequences of interactions between the bone marrow stroma and myeloma. *The Hematology Journal*.

